# Calebin A targets the HIF-1α/NF-κB pathway to suppress colorectal cancer cell migration

**DOI:** 10.3389/fphar.2023.1203436

**Published:** 2023-07-31

**Authors:** Aranka Brockmueller, Sosmitha Girisa, Mahzad Motallebi, Ajaikumar B. Kunnumakkara, Mehdi Shakibaei

**Affiliations:** ^1^ Chair of Vegetative Anatomy, Institute of Anatomy, Faculty of Medicine, Ludwig-Maximilians-University Munich, Munich, Germany; ^2^ Cancer Biology Laboratory, Department of Biosciences and Bioengineering, Indian Institute of Technology Guwahati, Guwahati, Assam, India; ^3^ Department of Biology, Yadegar-e-Imam Khomeini Shahr-e-Rey Branch, Islamic Azad University, Tehran, Iran

**Keywords:** Calebin A, 3D alginate, tumor microenvironment, CRC migration, HIF-1α, NF-κB, neoangiogenesis, apoptosis

## Abstract

**Background:** Hypoxia-inducible factor-1α (HIF-1α) is one of the major tumor-associated transcription factors modulating numerous tumor properties such as tumor cell metabolism, survival, proliferation, angiogenesis, and metastasis. Calebin A (CA), a compound derived from turmeric, is known for its anti-cancer activity through modulation of the NF-κB pathway. However, its impact on HIF-1α in colorectal cancer (CRC) cell migration is unknown.

**Methods:** Human CRC cells (HCT-116) in 3D alginate and monolayer multicellular TME (fibroblasts/T lymphocytes) were subjected to CA or the HIF-1α inhibitor to explore the efficacy of CA on TME-induced inflammation, migration, and tumor malignancy.

**Results:** CA significantly inhibited TME-promoted proliferation and migration of HCT-116 cells, similar to the HIF-1α inhibitor. Colony formation, toluidine blue staining, and immunolabeling showed that CA inhibited the migration of HCT-116 cells partly by inhibiting HIF-1α, which is critical for CRC cell viability, and these observations were confirmed by electron microscopy. In addition, Western blot analysis confirmed that CA inhibited TME-initiated expression of HIF-1α and biomarkers of metastatic factors (such as NF-κB, β1-integrin, and VEGF), and promoted apoptosis (caspase-3), in a manner comparable to the HIF-1α inhibitor. Finally, TME induced a purposeful pairing between HIF-1α and NF-κB, suggesting that the synergistic interplay between the two tumor-associated transcription factors is essential for CRC cell malignancy and migration and that CA silences these factors in tandem.

**Conclusion:** These results shed light on a novel regulatory modulation of CA signaling in CRC cell migration, partially via HIF-1α/NF-κB with potentially relevant implications for cancer therapy.

## Graphical Abstract

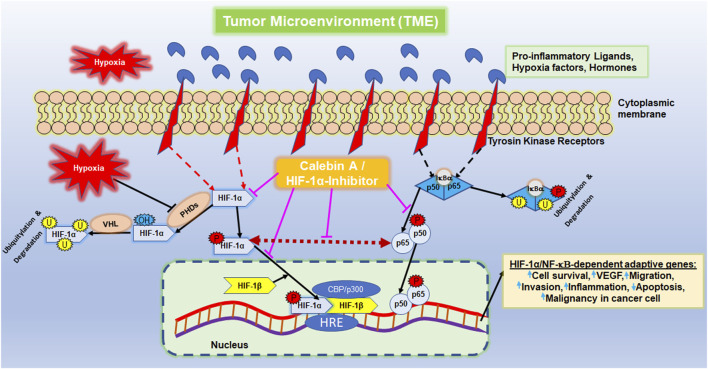

## 1 Introduction

Advanced colorectal cancer (CRC), defined as metastatic neoplasia of the colon or rectal epithelium, represents a global health problem. A statistical survey of patient data from 185 countries showed that in 2020, CRC was responsible for 10% of the 19.3 million new cancer cases and 9.4% of the 10 million cancer-related deaths (Sung et al., 2021). In particular, increasing numbers of cases are now observed among younger people, which can probably be linked to modern lifestyle factors (Sung et al., 2021). In general, the disease is characterized by high aggressiveness, resulting in high metastasis rates (Miller et al., 2022). Hereby, vital tumor cells with inflammation-facilitated and epithelial–mesenchymal transition (EMT)-triggered migratory capacity, as well as neoangiogenesis after tumor cell colonization, are requirements for metastasis development (Welch and Hurst, 2019). These events are closely interconnected and mutually reinforcing. For instance, it is well established that the transcription factor nuclear factor-κB (NF-κB) in CRC cells is not only the central wheel in escalating inflammation cascades but also its increased expression explicitly promotes EMT, resulting in an enhanced migratory ability (El-Ashmawy et al., 2019). Consistent with this finding, the prevention of CRC metastasis including morphological changes and increased cell motility has been demonstrated by NF-κB inhibition (Kim et al., 2017).

Interestingly, CRC cell migration and invasion were also promoted by angiogenesis-supporting transcription factor ‘hypoxia-inducible factor’ (HIF)-1α (Kim et al., 2017), which is, in turn, initiated by the inflammatory NF-κB-activated milieu in a tumor microenvironment (TME) (Buhrmann et al., 2020c). HIF-1α is among the most important transcriptional regulators associated with tumors, and its activity increases in hypoxia to initiate the fulfillment of oxygen demand. For this purpose, the hydroxylation of HIF-1α is inhibited, leading to the activation of erythropoietin and vascular endothelial growth factor (VEGF). On the whole, it affects the function of several transcription factors and modulates a number of different tumor functions, including cell metabolism, immune response, survival, proliferation, angiogenesis, and metastasis. For this reason, HIF-1α activation is largely responsible for poor treatment outcomes. HIF-1α is known to play an important role in hypoxia-induced angiogenesis, metastasis (Xing et al., 2011; Bocca et al., 2014), EMT and invasion of cancer cells (Jo et al., 2009; Xing et al., 2011; Bocca et al., 2014; Zhang et al., 2021; Zhang et al., 2022), and also in inducing conventional drug-resistant properties (Yu et al., 2012), thus playing a central role in tumor cell malignancy. Moreover, as the success of targeted monotherapy is uncertain, scientists have aimed in identifying agents that could target and interfere with multiple subcellular cell signaling pathways.

Numerous natural products are being considered for research and production of new safe and reliable substances for cancer prevention. The importance of different natural supplements such as polyphenols and flavonoids for cancer prevention has been extensively researched, and there is much circumstantial evidence that a relatively high intake of fruits and plant foods is associated with a reduced risk of CRC (Lee et al., 2020; Wang et al., 2020; Ahmad et al., 2022; Amintas et al., 2022; Mahmod et al., 2022; Rodríguez-García and Gutiérrez-Santiago, 2023). It has already been reported that these natural compounds are able to influence gene expression and their end products with their versatile modulatory signaling pathways, which allows them to regulate many events in the cell, such as the cell cycle, differentiation, survival, migration, and apoptosis (Shweiki et al., 1992; Dashwood et al., 1999; Latruffe et al., 2002; Mohamed et al., 2004; Shen et al., 2007; Pan et al., 2011; Costea et al., 2018; Pérez-Escalante et al., 2018; Wang et al., 2020). In addition, overproduction of pro-inflammatory transcription factors such as NF-κB, STAT3, and HIF-1α and cytokines such as IL-1β and TNF-α, which are important risk factors for colitis and the development of CRC, has been demonstrated more frequently, especially in the colon due to recurrent inflammation, which could be inhibited by various phytochemicals due to their anti-inflammatory effects, leading to CRC prevention (Gupta et al., 2010; Ma et al., 2017; Fang et al., 2018; Qin et al., 2022; Zeng et al., 2022).

Calebin A (CA) is a natural polyphenol, isolated from rhizomes of the Asian plants *Curcuma longa* and *Curcuma caesia* (Majeed et al., 2019). This curcuminoid component (Majeed et al., 2019) has shown valuable properties for human health in initial preclinical trials by protecting various organ systems such as the nervous system (Kim and Kim, 2001) and modulating the oxidative and metabolic states (Oliveira et al., 2015). Furthermore, early indications of a strong anti-inflammatory potential were found, especially via the regulation of NF-κB (Tyagi et al., 2017), which is mainly responsible for inflammation, with high relevance in acute and chronic inflammations, and resulting cancers (Fauve et al., 1974; Libermann and Baltimore, 1990; Neurath et al., 1996). Taking advantage of this, CA averted NF-κB activation in cells of multiple myeloma and head and neck cancer (Tyagi et al., 2017), leading to cancer cell growth inhibition. Moreover, CA suppressed the proliferation of cells from malignant peripheral nerve sheath tumors in both *in vitro* and *in vivo* conditions, resulting in tumor size reduction in a xenograft mouse model (Lee et al., 2019). Furthermore, CA treatment contributed to inhibiting proliferation and metastasis while stimulating apoptosis in various CRC cell lines via the NF-κB pathway (Tyagi et al., 2017; Buhrmann et al., 2019; Buhrmann et al., 2020b). This even led to an increase in the effect of the CRC standard chemotherapeutic 5-fluorouracil (5-FU) in largely chemoresistant cells (Buhrmann et al., 2020a).

To the best of our knowledge, it has not yet been described whether treatment of CRC cells with CA affects their vascularization abilities including the expression of HIF-1α. Therefore, we investigated the effects of CA on HCT-116 CRC cells in a 3D-TME *in vitro* cultivation, focusing on a possible functional link between the tumor-promoting transcription factors NF-κB and HIF-1α.

## 2 Materials and methods

### 2.1 Antibodies and additives

The monoclonal antibody against HIF-1α (sc-13515), VEGF (sc-7269), and normal mouse IgG was obtained from Santa Cruz (Dallas, Texas, United States). Monoclonal anti-phospho-p65-NF-κB (#MAB7226), anti-p65-NF-κB (#MAB5078), anti-PARP (#MAB8095), and polyclonal anti-cleaved-caspase-3 (#AF835) were purchased from R&D Systems (Heidelberg, Germany). The monoclonal anti-β1-integrin (#14-0299-82) was obtained from Thermo Fisher Scientific (Langenselbold, Germany). Monoclonal anti-β-actin (#A4700), BMS-345541 (IKK inhibitor), DAPI, MTT reagent, toluidine blue, alginate, Fluoromount, and 2-mercaptoethanol were obtained from Sigma-Aldrich (Taufkirchen, Germany). Secondary Western blot antibodies (alkaline phosphatase-linked) were purchased from EMD Millipore (Schwalbach, Germany), and secondary immunofluorescence antibodies (rhodamine-coupled) were obtained from Dianova (Hamburg, Germany). HIF-1α inhibitor (PX-478), a specific suppressor of HIF-1α levels in cancer cells, was bought from Cayman Chemical (Ann Arbor, Michigan, United States). Epon was bought from Plano (Marburg, Germany). CA was donated by Sabinsa Corporation (East Windsor, New Jersey, United States). It was prepared as a 5,000 µM stock solution in dimethyl sulfoxide (DMSO) and finally diluted in the cell culture medium without exceeding 0.1% DMSO during the experiments.

### 2.2 Cell cultivation

The following cells were used in the presented studies: HCT-116 CRC cells (human) and MRC-5 fibroblasts (human) from the European Collection of Cell Cultures (Salisbury, United Kingdom), and Jurkat T lymphocytes (human) from the Leibniz Institute (Braunschweig, Germany). HCT-116 and MRC-5 grow as a monolayer, and Jurkat floats in the cell culture medium. All cell lines were cultivated in T75 cell culture flasks at 37°C and 5% CO_2_. The DMEM F-12 (1:1) cell culture medium from Sigma-Aldrich (Taufkirchen, Germany) was supplemented with 3% fetal bovine serum (FBS, serum-starved) or 10% FBS, 1,2% penicillin/streptomycin, 1% L-glutamine, 1% ascorbic acid, 1% essential amino acids, and 0. 5% amphotericin B, as used previously (Brockmueller et al., 2022b).

### 2.3 3D alginate matrix model

To set up an alginate model similar to previous attempts (Brockmueller et al., 2022c), CRC cells were grown as a monolayer in T75 cell culture flasks until 70%–80% confluency. Passage 2 or 3 was counted (1 million) and resuspended in alginate (1 ml, 2% alginate in a 0.15 M NaCl solution). The suspension was dropped into a CaCl_2_ solution and polymerized for 10 min. Subsequently, alginate drops were washed three times in Hank’s salt solution and twice in a cell culture medium containing 10% FBS and then incubated for 30 min in the cell culture medium. The experiments were carried out as 3D cultures, with or without a multicellular TME and with or without CA (1, 2, and 5 µM) or the HIF-1α inhibitor (1, 10, 20, and 30 µM). During 10–14 days of experimental run time, the cell culture medium contained 3% FBS to superficially assess the effect of additives.

### 2.4 Multicellular tumor microenvironment

The TME was composed of CRC cells embedded in an alginate matrix, fibroblasts grown as a monolayer on the bottom, and T-lymphocytes floated in a cell culture medium as a 3D cultivation model in 12-well plates. This construction mimicked a pro-inflammatory and *vivo*-like cancerous body situation by multicellular cultivation *in vitro*, avoiding animal testing as already been established (Buhrmann et al., 2020c).

### 2.5 Colony formation investigation

Growth progression of CRC cells in the alginate drops was observed for 10–14 days using an Axiovert 40 CFL phase contrast microscope (Zeiss, Oberkochen, Germany); photographs were taken, and pictures were digitally stored, as mentioned previously (Shakibaei and De Souza, 1997; Shakibaei et al., 2015).

### 2.6 Invasion investigation

The CRC cells multiplied in the alginate drops and formed colonies that migrated out of the spheres and then settled on the ground of 12-well plates. After 10–14 days, the 12-well plates were fixed in a Karnovsky solution and stained with toluidine blue, which resulted in the appearance of the settled colonies. These colonies were clearly recognizable as large dark blue dots, while the fibroblast monolayer was stained only light blue so that the two cell types were clearly distinguishable. They were counted per well and averaged from each treatment in three independent trials, as already described (Shakibaei et al., 2015; Buhrmann et al., 2019).

### 2.7 MTT viability and cytotoxicity investigation

For measuring the CRC cell viability, HCT-116 cells were embedded in an alginate matrix, and after 10–14 days of treatment in a 3D culture as described previously, a MTT assay was carried out. Therefore, the CRC-alginate drops were removed with bent tweezers from the experimental 12-well plate and washed three times in Hank’s salt solution so that only HCT-116 cells were measured as explained previously in detail (Brockmueller et al., 2022c). Hereafter, the unpolluted CRC-alginate drops were dissolved in 55 mM sodium citrate and centrifuged. Alginate residues were removed, and HCT-116 cells were washed in Hank’s salt solution and resuspended in the MTT medium containing 3% FBS but without vitamin C and phenol red. A measure of 100 μl of the cell suspension and 10 µl of the MTT solution were distributed to each well of a 96-well plate. By admixing 100 µl of the MTT solubilization solution containing 10% Triton X-100/acidic isopropanol to each well, the reaction was terminated after 3 h. With a ‘Bio-Rad ELISA reader’ (Munich, Germany), the optical density (OD) was obtained at 550 nm.

### 2.8 Migration investigation

For the implementation of the migration assay, the 3D experimental set-up was modified. First, HCT-116 cells were seeded on small, round glass 12-well plates (80,000 cells/well) and grown in a cell culture medium containing 10% FBS. After 24 h, a wound incision was made in the grown CRC monolayer using a standard pipette tip (50–1,000 µl) from Eppendorf (Hamburg, Germany). The dissolved CRC cells were washed off with Hank’s salt solution, wells were replenished with the 3% FBS cell culture medium, and wound incisions were photographed using a phase contrast Axiovert 40 CFL microscope (Zeiss, Oberkochen, Germany). Now, the trial substances were added for 2 h, and then, the glass plates were located in a 3D-model, as already used previously (Buhrmann et al., 2021b). The 3D-environment was built in 6-well plates with each well containing a small steel bridge on which the CRC-glass plates were placed. Although the cells of the basal control were only surrounded by the cell culture medium, in TME treatment, there were also fibroblasts at the bottom of the 6-well plates and free-floating T lymphocytes. After 1–3 days, the CRC-glass plates were removed from the bridges with tweezers, wound incisions were observed in a 12-well plate, and the CRC-glass plates were placed back on the steel bridges. The wound incision width was recorded at three different positions on the respective light image, and the mean value was calculated. The extent of closure of wound incisions over a period of 3 days was calculated and compared with the control. After 2 days, the CRC-glass plates were washed with Hank’s salt solution, photographed again in a 12-well plate, methanol-fixed for immunolabeling, and frozen at −20°C.

### 2.9 Immunofluorescence investigation

Methanol-fixed CRC-glass plates from the 3D culture environment (detailed described in the ‘Migration assay’ section and earlier publications (Buhrmann et al., 2021b; Brockmueller et al., 2022b)) were defrosted, washed with Hank’s salt solution, and then processed with the Triton solution (0.5% in Hank’s salt solution) and bovine serum albumin solution (1% in Hank’s salt solution). For immunolabeling, the cells were incubated with the primary antibody (dilution 1:80) overnight in a moist chamber. The next day, the cells were incubated in the secondary (dilution 1:100) antibody for 90 min and stained with DAPI for 15 min. Finally, the CRC-glass plates were covered in Fluoromount and examined and photographed with a Leica DM 2000 microscope (Wetzlar, Germany), in accordance with the previous protocol (Brockmueller et al., 2022c).

### 2.10 Electron microscopic investigation

HCT-116 were 3D-cultivated on small, round glass plates equal to the process described in the ‘Migration assay’ and ‘Immunofluorescence’ sections. The CRC-glass plates were fixed in the Karnovsky solution (1 h) and then trans-filled into tubes by means of a cell scraper, followed by a fixation with osmium tetroxide (O_s_O_4_, 2 h). Afterward, they were dehydrated (ascending alcohol series) and embedded in Epon, as explained previously (Shakibaei and De Souza, 1997; Buhrmann et al., 2018). The samples were processed with Reichert-Jung Ultracut E (Darmstadt, Germany) and contrasted (2% uranyl acetate/lead citrate solution). The investigation of ultrastructural changes was carried out with a transmission electron microscope (TEM) 10 from Zeiss (Jena, Germany).

### 2.11 Western blot evaluation

Western blot samples were obtained from HCT-116 cells that had been grown in alginate drops for 10–14 days with or without treatment additives and with or without TME, as mentioned previously. The CRC-alginate drops were removed from experimental 12-well plates and washed in Hank’s salt solution to ensure that only HCT-116 cells were investigated. After that, CRC cells were dissolved from the alginate matrix with a sodium citrate solution, resuspended in lysis buffer, centrifuged for 30 min, and the supernatant was frozen at −80°C. The sample proteins were determined with a ‘Protein Quantification Kit’ from Interchim (Montlucon Cedex, France) and reduced with 2-mercaptoethanol. Subsequently, the samples were separated with a transblot apparatus from Bio-Rad (Munich, Germany) by SDS-PAGE, as described previously (Shakibaei et al., 2015; Brockmueller et al., 2022c). Nitrocellulose membranes from Fisher Scientific (Schwerte, Germany) were pre-incubated in blocking buffer for 2 h, incubated overnight with a primary antibody (dilution 1:10,000), and subsequently incubated with a secondary antibody (dilution 1:5,000) for 90 min. 1-Step™ NBT/BCIP from Fisher Scientific (Schwerte, Germany) was used to finalize, and densitometric evaluation was carried out via the ‘Quantity One’ program from Bio-Rad (Munich, Germany).

### 2.12 Immunoprecipitative investigation

HCT-116 samples were collected as explained in the ‘Western blot evaluation’ section and immunoprecipitated to study the functional link between NF-κB and HIF-1α signaling. For this purpose, the samples were incubated with 25 µl IgG serum (normal mouse or rabbit) and *Staphylococcus aureus* to preclear. Hereafter, they were treated with anti-p65-NF-κB (primary antibody, 4°C/2 h) and processed with *S. aureus* (4°C/1 h), as carried out previously (Buhrmann et al., 2014). The SDS-PAGE separation was performed, as described in the ‘Western blot evaluation’ section with HIF-1α as an antibody.

### 2.13 Statistical analysis

Investigations were performed as three independent experiments, and data were evaluated by unpaired Student’s t-test and ANOVA (one-way) by a *post hoc* test to contrast each group’s parameters. A significant difference was indicated by *p*-values less than 0.05.

## 3 Results

### 3.1 Calebin A suppresses CRC cell viability, proliferation, and metastatic capacity, similar to the HIF-1α inhibitor

**FIGURE 1:**
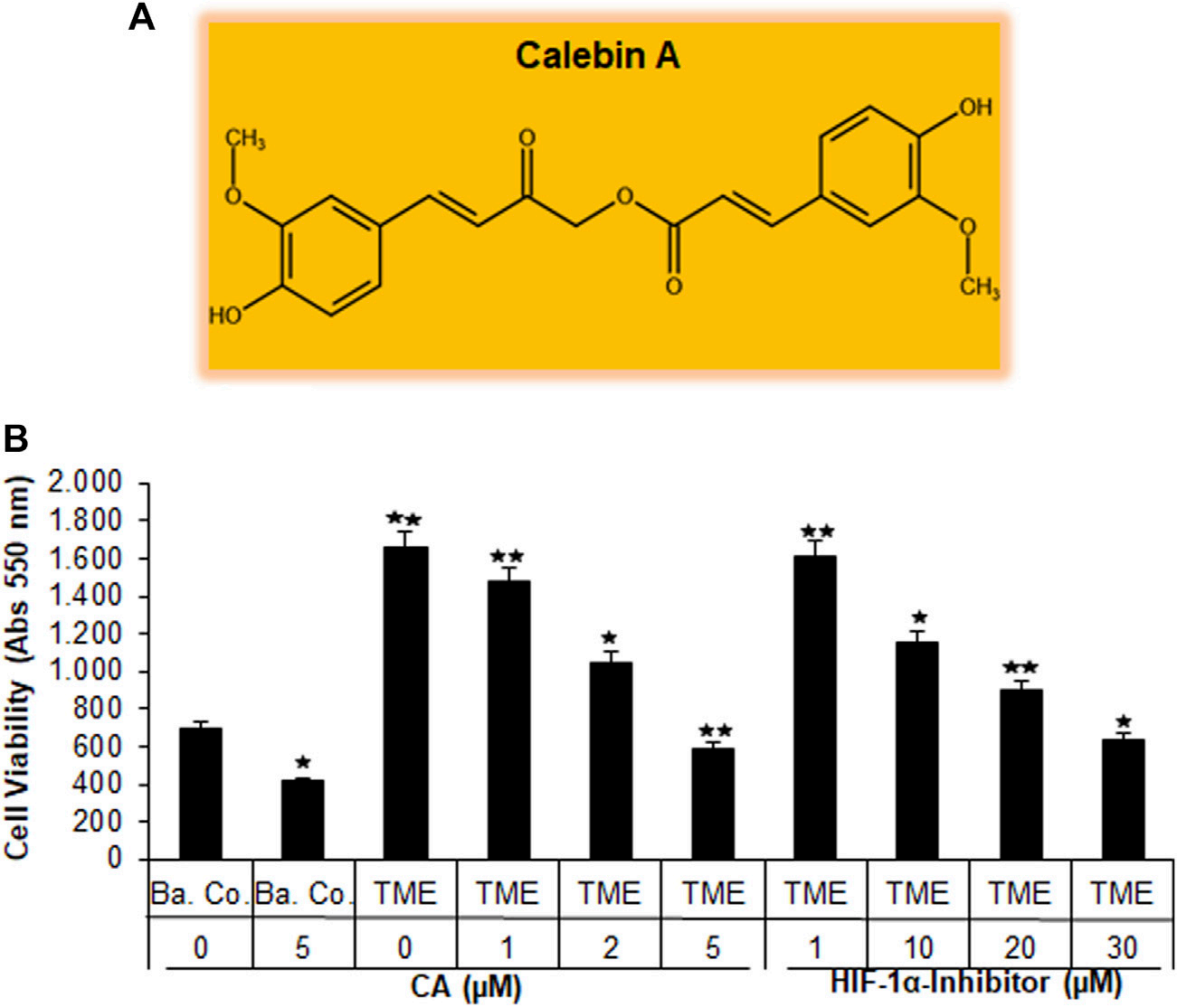
Effect of Calebin A or the HIF-1α inhibitor on CRC cell survival. **(A)** The chemical formula of CA. **(B)** X-axis: HCT-116 cells in alginate drops either self (basal control, Ba. Co.) or in the TME were, first, not treated or, second, exposed to a series of doses of CA (1, 2, and 5 µM) or the HIF-1α inhibitor (1, 10, 20, and 30 µM). Y-axis: cell survival was monitored by MTT evaluation at 550 nm OD. Compared with Ba.Co.: *p < 0.05 and **p < 0.01.

Recently, angiogenesis inhibitors such as bevacizumab or ramucirumab have been integrated into CRC treatment, especially at an advanced stage of the disease. Both are monoclonal antibodies that inhibit VEGF through receptor binding, leading to the suppression of new blood vessel formation and thus to a bottleneck in tumor supply (Hurwitz et al., 2005; Garcia-Carbonero et al., 2014). This led us to consider the emerging multi-targeted-phytopharmacon Calebin A (Figure 1A) for its anti-neovascularizing properties in CRC cells. For this purpose, we chose the human CRC cell line HCT-116 and compared the effect of CA with that of a specific HIF-1α inhibitor in 3D alginate CRC culture models. Therefore, the HCT-116 cells were embedded in the alginate matrix, as described in the ‘Material and methods’ section, and it was treated and examined as follows: first, a basal control without the TME, followed by a TME control (including Jurkat and MRC-5 cells as explained in Material and methods) without additives and concentration-dependent treatments of the TME with CA (1, 2, and 5 µM) or the HIF-1α inhibitor (1, 10, 20, and 30 µM), was taken. After completion of the experiments, the CRC cell’s viability, proliferation, and metastatic capacity were evaluated in the following section.

#### 3.1.1 Inhibition of CRC cell viability and toxicity

Initially, the vitality of the HCT-116 cells that have been separated from the 3D alginate matrix was compared and evaluated by the MTT assay (Figure 1B). A basal control culture confirmed overall cell viability without the influence of the pro-inflammatory, multicellular TME. Interestingly, the addition of 5 µM CA to the basal control cultures significantly reduced the number of viable HCT-116 cells by more than one-third. The TME made a remarkable difference in this experiment: when the CRC cells were grown on it (as a control without the addition of any other substances), the number of viable cells was more than twice as high as in the basal control cultures. The subsequent treatment of HCT-116 cells in the TME with an ascending concentration series of CA (1, 2, and 5 µM) led to a continuous decrease in their cell viability. This was particularly significant with the addition of 5 μM CA, which reduced the number of viable cells by more than half when compared to TME control cultures (Figure 1B). Subsequently and comparatively, the concentration-dependent treatment of a specific HIF-1α inhibitor (1, 10, 20, and 30 µM) was investigated. Thereby, the number of viable CRC cells was almost halved with the addition of 20 µM of the HIF-1α inhibitor, and only the addition of 30 µM HIF-1α inhibitor produced a viability-reducing effect comparable to that of 5 µM CA (Figure 1B).

Overall, these results suggest that CA suppressed the cell viability of CRC cells in a manner similar to that of the HIF-1α inhibitor.

#### 3.1.2 Inhibition of CRC cell proliferation and colony formation

**FIGURE 2:**
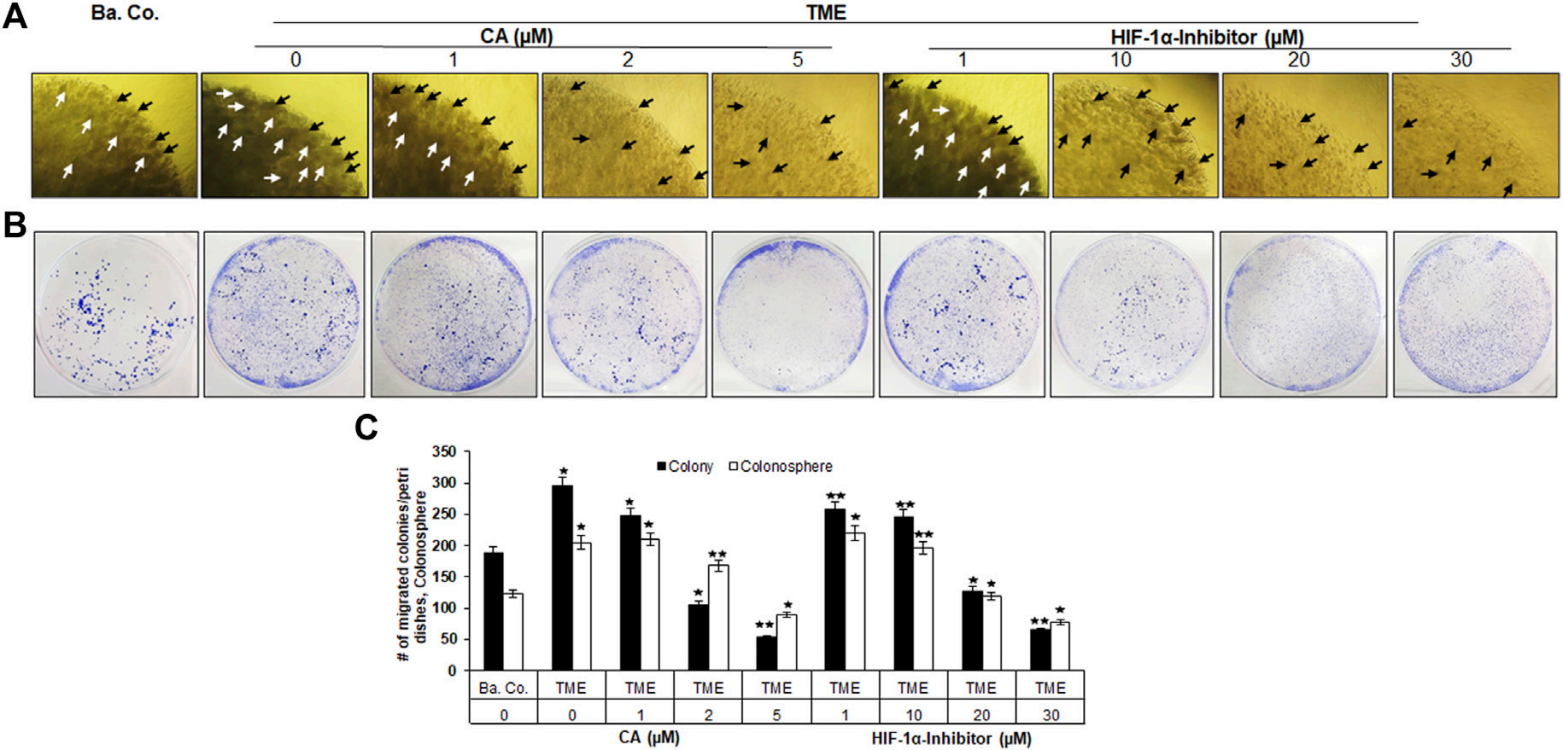
Effect of Calebin A or the HIF-1α inhibitor on colony formation and invasion properties of CRC cells. HCT-116 cells were embedded in the alginate matrix and treated as follows: basal control (Ba. Co.) or TME without additives or TME with the dose-dependent concentration of CA (1, 2, and 5 µM) or the HIF-1α inhibitor (1, 10, 20, and 30 µM). The evaluation was performed by **(A)** colony formation observation: arrows represent the formed CRC cell colonies in the alginate matrix (black/white arrows are synonymous, alternated for better visibility) and **(B)** invasion investigation: invaded CRC cell colonies settled on the bottom of the well plate were stained with toluidine blue. **(C)** Statistic report of **(A)** and **(B)**. X-axis: treatments; y-axis: number of formed CRC cell colonies (white bars) and number of settled CRC cell colonies (black bars). Relative to Ba.Co.: *p < 0.05 and **p < 0.01.

Before the HCT-116 cells were removed from the alginate drops, their growth behavior in the basal control, TME control, and TME under the influence of CA (1, 2, and 5 µM) or the HIF-1α inhibitor (1, 10, 20, and 30 µM) was observed in detail, as described in colony formation investigation. Viewing under the light microscope, visible, treatment-dependent differences in proliferation and colony formation behavior were documented (Figure 2A). In the inflammation-stimulated TME control cultures, significantly more colonospheres had formed (Figure 2A, arrows) than those in the basal control cultures. This high proliferation rate also persisted with the addition of 1 µM CA and decreased slightly at 2 µM CA. TME treatment with 5 μM CA finally reduced the number of colonospheres ready to emigrate by approximately 50% (Figures 2A, C, white bars). Moreover, an addition of the HIF-1α inhibitor also reduced the number of CRC cell colonospheres in a concentration-dependent manner, whereby treatment with 30 µM had a similar proliferation-inhibiting effect as a form of treatment with 5 µM CA (Figures 2A, C).

Altogether, this observation was consistent with the previously described MTT assay result and highlights the selective and precise blockade of colony formation as one of the many anti-tumor mechanisms in CRC-TME by CA, similar to the suppression of colony formation by targeted HIF-1α inhibitors, which shows at least in part a HIF-1α protein dependence.

#### 3.1.3 Inhibition of CRC cell invasion

In parallel to the proliferation and colony formation observation, it was also analyzed how many cells were finally settled as colonies at the bottom of the well plates, as described in the Materials and methods section, symbolizing the metastatic potential of HCT-116 cells. With this aim, the well plates were stained with toluidine blue after the end of the experiment, whereby the colonies appear as distinctive dark blue dots (Figure 2B). The statistical evaluation (Figure 2C, black bars) revealed that the multicellular TME promoted the invasion of CRC cells and, thus, the colonies by more than one-third compared to the basal control cultures. The higher the dosage of CA (1, 2, and 5 µM) added to the TME cultures, the fewer colonies settled at the bottom of the experimental well plates. Interestingly, treatment of the alginate-embedded CRC cells with 5 µM CA reduced the invasion and colony formation and inhibited the metastatic capacity by more than 80%. In comparison, the addition of the HIF-1α inhibitor (1, 10, 20, and 30 µM) also caused a concentration-dependent decrease in the colony count. To sum up, the evaluations by the MTT viability test, colony formation observation, and invasion investigation confirmed each other and provided consistent evidence that the effect of 5 µM CA is similar to that of the 30 µM HIF-1α inhibitor in inhibiting viability, proliferation, and the metastatic capacity of HCT-116 CRC cells.

### 3.2 Calebin A suppresses TME-induced migration of CRC cells similar to the HIF-1α inhibitor

To study the migration capacity of the CRC cells in more detail, we initiated a wound-healing assay (migration assay). For this purpose, the HCT-116 cells were grown on round glass plates and, after monolayer incision, cultivated on small steel bridges in a TME culture. Wound healing was measured by an average reduction in the area between the wound edges at the respective times and dosages.

**FIGURE 3:**
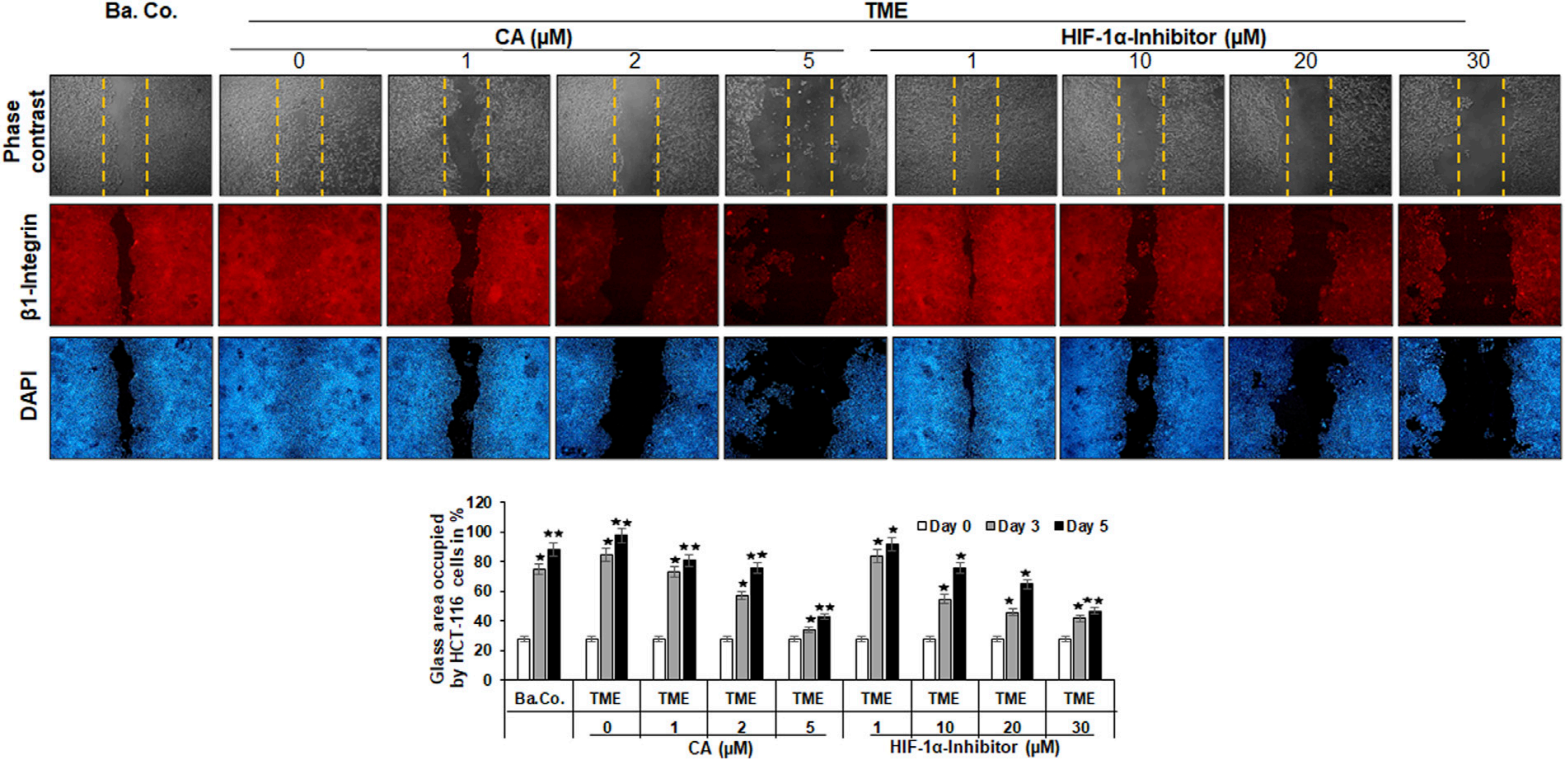
Effect of Calebin A or the HIF-1α inhibitor on the migration of CRC cells. The migration of HCT-116 cells on a wound incision-exposed surface in a CRC cell monolayer on glass plates (procedure described in detail in the ‘Migration investigation’ section, Material and methods) was carried out under different conditions: first, a treatment-free basal control (Ba. Co.) and TME control (TME). Second, the TME was treated with 1, 2, and 5 µM CA or 1, 10, 20, and 30 µM HIF-1α inhibitor. Analysis was carried out by phase contrast microscopy (upper row; yellow dashed lines represent the distance of the made wound incision), immunofluorescence (middle row; red labeling represents the β1-integrin), and DAPI staining (lower row; blue marking shows DAPI staining). The images shown represent the CRC cells at treatment day 5. The bar chart shows different CRC cell treatments on the x-axis and the measured glass plate area occupied by HCT-116 cells (%) on the y-axis in the course of the trial. White bars represent day 0, monolayer with standardized wound incision. Gray bars (day 3) and black bars (day 5) represent CRC cell migration in the course of treatment. Statistics with n = 3: *p < 0.05 and **p < 0.01 related to basal control.

As shown in Figure 3, different treatments (basal control, TME control, and TME with CA (1, 2, and 5 µM) and TME with 1, 10, 20, and 30 µM HIF-1α inhibitors were evaluated by phase contrast microscopy (upper row, gray) and β1-integrin immunolabeling (middle row, red), as a cell surface receptor (migration-biomarker), and DAPI staining (lower row, blue), as a marker for vital cells.

The first striking observation was the tumor-promoting effect of the TME, noticeable in a dense influx with complete closure of the wound incision, while a narrow wound incision opening remained in the basal control cultures at the end of the experiment. Remarkably, an addition of CA (1, 2, and 5 µM) to the TME showed two concentration-dependent effects: a) it significantly prevented the migration of the incision (an inhibition of more than 50% compared to the addition-free TME) and b) the expression of the cell surface receptor β1-integrin was inhibited, visible by weaker immunolabeling while the DAPI-staining remained strong (Figure 3). More interestingly, a similar, migration-inhibiting and β1-integrin-weakening effect was also presented by the HIF-1α inhibitor (1, 10, 20, and 30 µM), and in this experiment, these effects were also observed at significantly higher concentrations (Figure 3).

In addition to the images shown, representing the fifth, final day of the trial, the treatment-dependent migratory dynamics were measured and statistically processed during the course of the trial. The white bars show a uniform initial situation of the glass plates after the monolayer wound incision, in which approximately 28% of the glass surface was occupied by HCT-116 cells. The gray bars mark the intermediate state of progress on day 3, and the black bars represent the evaluation of the final situation on day 5 (Figure 3). In summary, the multicellular 3D-TME effectively stimulated the CRC cells in a tumor-promoting manner, and both CA and the HIF-1α inhibitor suppressed their migratory propensity in a concentration-dependent manner. The dose of CA required to achieve a similar effect was lower than that of the HIF-1α inhibitor so that the differences observed in the alginate matrix were also reflected in HCT-116 monolayer cultures. Moreover, the observation of β1-integrin immunolabeling weakened by CA or the HIF-1α inhibitor could indicate the use of the β1-integrin receptors by both substances for migration inhibition and offers a future research question.

### 3.3 Calebin A suppresses intranuclear activation of HIF-1α in CRC cells similar to the HIF-1α inhibitor

**FIGURE 4:**
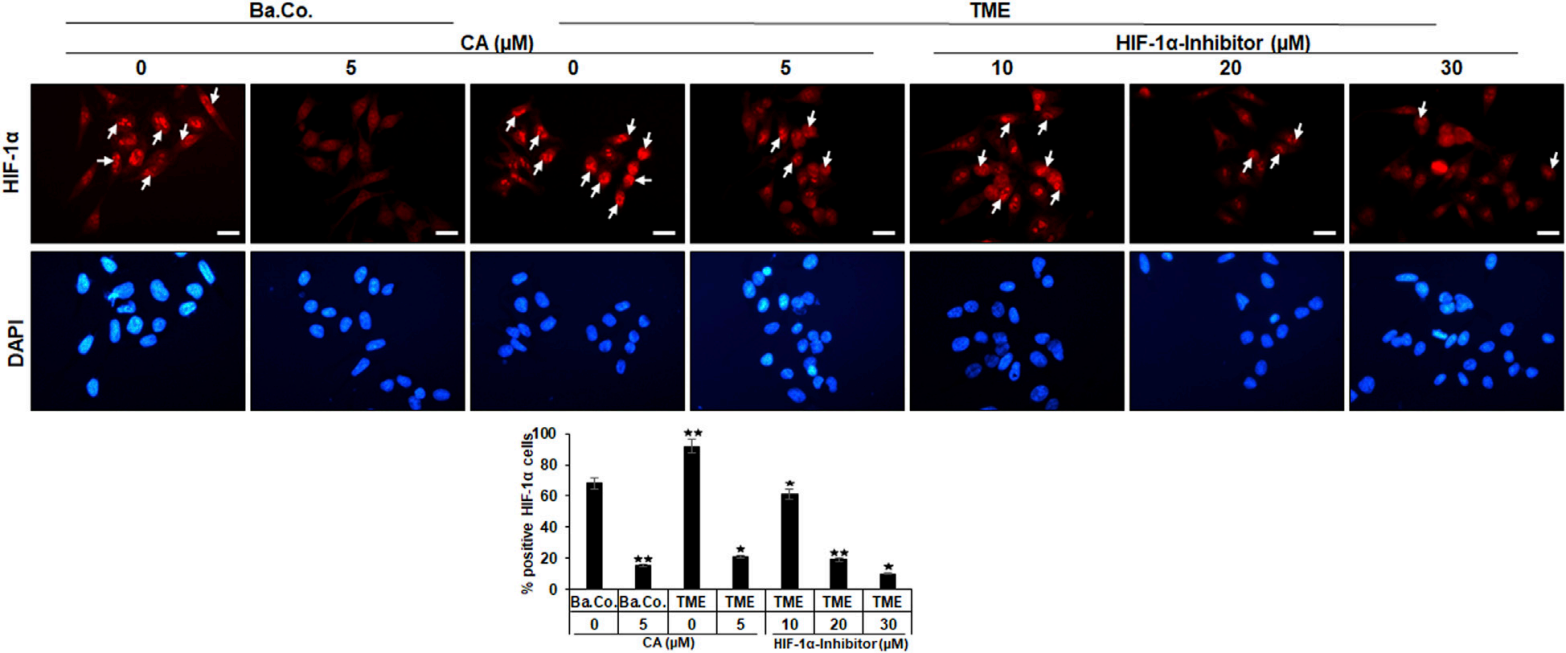
Effect of Calebin A or the HIF-1α inhibitor on immunocytochemical HIF-1α localization in CRC cells. HCT-116 cells grown on glass plates were incubated without TME as a basal control (Ba. Co.) or with the TME and with/without CA (5 µM) or HIF-1α inhibitor (10, 20, and 30 µM) addition. CRC cells were immunolabeled by anti-HIF-1α (upper row; red labeled; white arrows), stained with DAPI (lower row: blue marked), and investigated by immunofluorescence microscopy. Scale bars = 30 µm. Determination of positively labeled HIF-1α cells (white arrows) was performed by counting 400–500 cells from 15 microscopic areas. Statistics with n = 3: *p < 0.05 and **p < 0.01 related to the basal control.

Another investigation in the 3D-monolayer model was the immunofluorescence examination, as described in the “Material and methods” section. In this experiment, a basal control with or without CA (5 µM) was compared with HCT-116 cells in the TME without additives or with CA (5 µM) or with an addition of the HIF-1α inhibitor (10, 20, and 30 µM). Afterward, the CRC cells were immunolabeled with a monoclonal antibody against HIF-1α (Figure 4, upper row, red) for detection of the activated protein and were DAPI-stained (Figure 4, lower row, blue) to reinsure the vitality of the HCT-116 cells.

In both, basal control and TME control, the CRC cell nuclei were significantly immunomarked (Figure 4, upper row, white arrows) in accordance with an active, intranuclear localization of the HIF-1α protein. However, treatment with CA significantly reduced the number of positively labeled HCT-116 cells with HIF-1α, more effectively as compared to basal control and TME control. Not surprisingly, treatment with the HIF-1α inhibitor also inhibited the expression of the HIF-1α protein, this time with greater efficiency than CA. As shown in Figure 4, from a concentration of 20 µM HIF-1α inhibitor, a stronger HIF-1α inhibition was achieved than that by the CRC treatment with 5 µM CA. Overall, these results confirmed a) the specific efficacy of the synthetic HIF-1α inhibitor and b) the great potential of a HIF-1α inhibitor-like effect of the natural component CA in HCT-116 CRC cells.

### 3.4 Calebin A suppresses EMT morphology in CRC cells similar to the HIF-1α inhibitor

**FIGURE 5:**
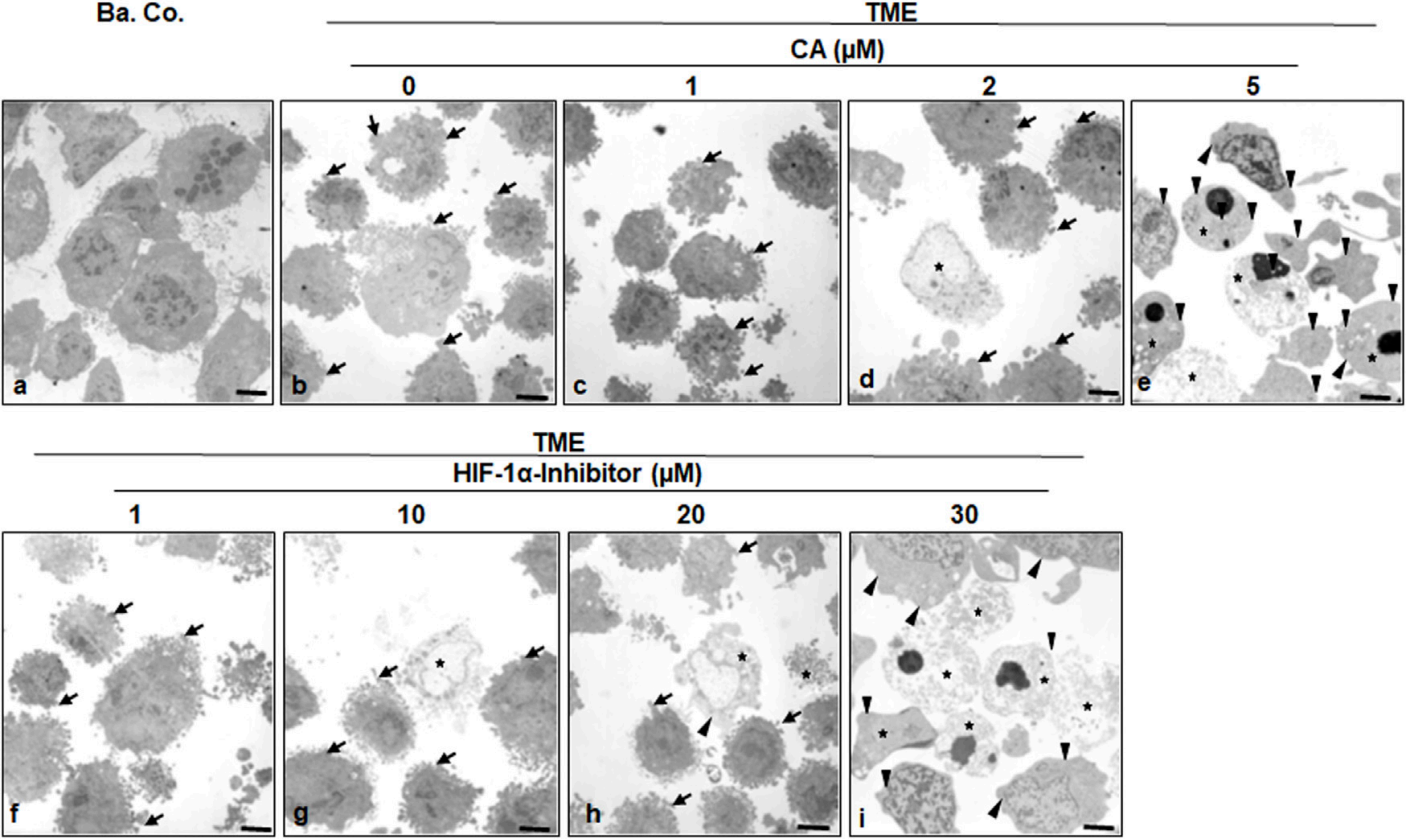
Effect of Calebin A or the HIF-1α inhibitor on ultrastructural morphology of CRC cells. HCT-116 cells were grown on glass plates and handled treatment-free as a basal control (Ba. Co.) or TME control, or TME was treated with a concentration-dependent series of CA (1, 2, and 5 µM) or the HIF-1α inhibitor (1, 10, 20, and 30 µM). The samples presented were examined by TEM. Symbols: arrows represent active pseudopodia, arrowheads represent the epithelial cell surface, and stars represent apoptotic bodies; scale bars = 1 µm.

To specify the morphological influences of CA (1, 2, and 5 µM) and the HIF-1α inhibitor (1, 10, 20, and 30 µM) leading on EMT, HCT-116 cells in basal control or in the TME, were compared and examined at the ultrastructural level. Therefore, the CRC cells were also grown in the already mentioned monolayer model in the TME (in-depth description in Materials and methods) and subsequently evaluated by transmission electron microscopy (TEM, Figure 5). Compared to the CRC cells in the basal control (Figure 5A), inflammation-stimulated HCT-116 cells showed much more active pseudopodia (black arrows) in the TME control (Figure 5B), underlining their strong migration tendency. We observed a decrease in these active pseudopodia at an addition of 1 µM CA (Figure 5C) to the TME, and furthermore, apoptotic bodies (black stars) appeared from an addition of 2 µM CA (Figure 5D). On a highly interesting note, TME treatment with 5 µM CA (Figure 5E) induced two significant changes: a) the mesenchymal, pseudopodia-rich surface of the HCT-116 cells became smooth with an epithelial, migration-inhibited characteristic (black arrowheads) and, moreover, b) the number of visible apoptotic bodies increased. Again, CA’s effects were compared with those of the HIF-1α inhibitor. Its addition by 1 µM did not result in a significant TME change because the CRC cells remained pseudopodia-active (Figure 5F). Then, treatment with 2 µM HIF-1α inhibitor (Figure 5G) caused, first, apoptotic bodies and an effect comparable to the treatment of 2 µM CA (Figure 5D). From a concentration of the 20 µM HIF-1α inhibitor (Figure 5H) onward, there was an increase in apoptotic bodies, while the activity of mesenchymal pseudopodia was reduced and, at the same time, the first epithelial-tending surfaces became observable. Finally, an addition of the 30 µM HIF-1α inhibitor (Figure 5I) resulted in a cell morphology comparable to that of the changes caused by CA (5 μM, Figure 5E), with numerous apoptotic bodies and predominantly smooth epithelial surfaces. Altogether, similar to the HIF-1α inhibitor, CA efficiently induced apoptosis in HCT-116 cells, smoothed their surface to epithelial morphology, and significantly reduced their migration propensity and thus their metastatic ability.

### 3.5 Calebin A suppresses neoangiogenesis, vascularization, inflammation, and migration, and promotes apoptosis in CRC cells, similar to the HIF-1α inhibitor

In the next step, the expression of central proteins representing neoangiogenesis (HIF-1α), vascularization (VEGF), inflammation (NF-κB), migration (β1-integrin), and apoptosis (cleaved-caspase-3) was investigated, taking into account the molecular conditions. For this purpose, HCT-116 cells were cultivated in the alginate matrix TME model under different treatments: basal control, TME control, and TME with CA (1, 2, and 5 µM) or the HIF-1α inhibitor (1, 10, 20, and 30 µM). At length, the CRC cells were removed from alginate and processed, as well as analyzed by Western blot as described in the ‘Material and methods’ section.

**FIGURE 6:**
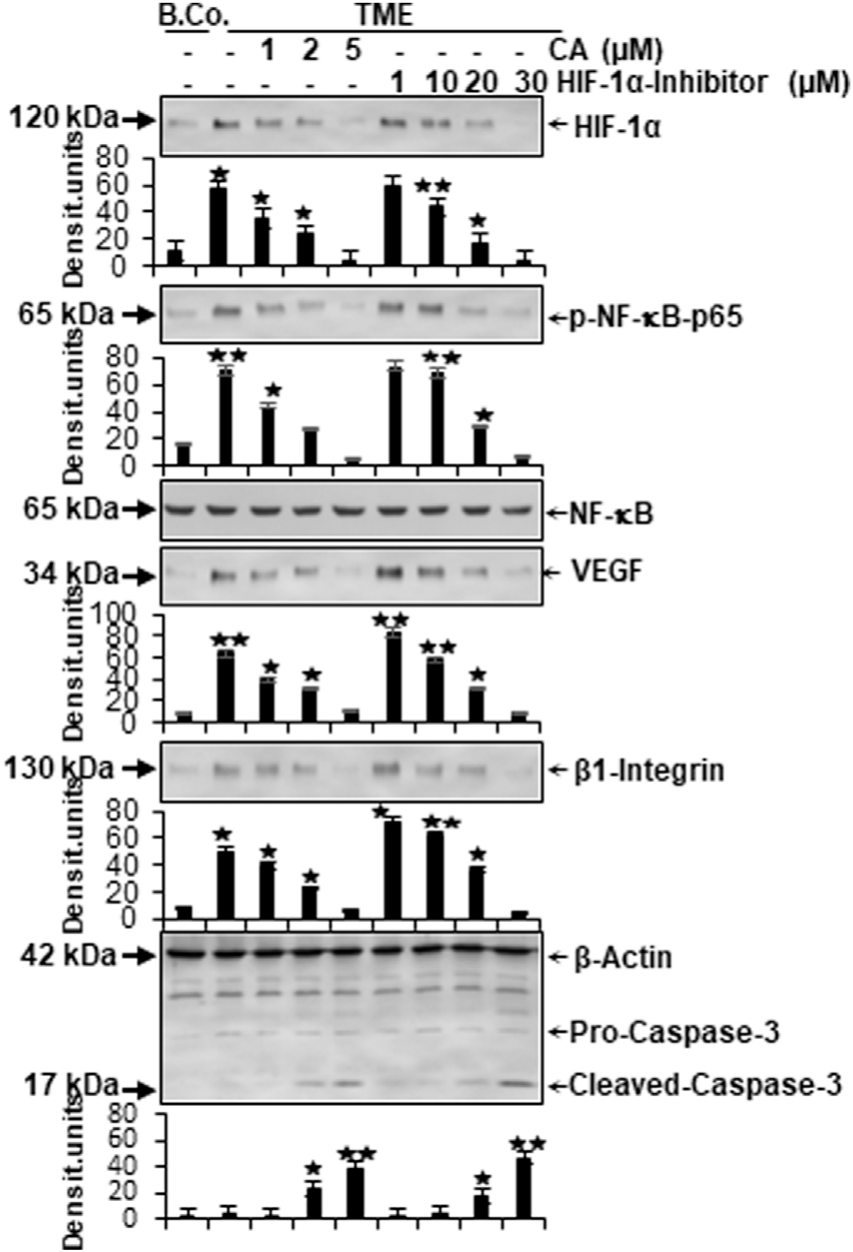
Effect of Calebin A or the HIF-1α inhibitor on vascularizing, inflammatory, metastatic, and apoptotic parameters. HCT-116 samples were isolated from the alginate matrix and assessed by Western blotting. X-axis shows the treatments carried out: basal control (B.Co.) or TME control, TME enriched with CA (1, 2, and 5 µM), or the HIF-1α inhibitor (1, 10, 20, and 30 µM). Y-axis oriented to densitometric units. In relation to Ba. Co.: *p < 0.05 and **p < 0.01. Antibodies against HIF-1α, phosphorylated/unphosphorylated NF-κB, VEGF, β1-integrin, and caspase-3 were used. β-actin served as a loading control.

Focusing on neoangiogenesis and vascularization, we first looked at the parameters associated with it. Both HIF-1α and VEGF were more highly expressed in the multicellular TME than in the basal control cultures. Interestingly, concentration-dependent treatment with CA or the HIF-1α inhibitor reduced the expression of both parameters significantly in CRC cells, reaching a minimum at 5 µM CA or 30 µM HIF-1α inhibitor. Thus, their dynamics confirmed each other (Figure 6). An escalating inflammatory NF-κB cascade represents a known fundamental problem in promoting tumorigenesis (Fauve et al., 1974; Tyagi et al., 2017), so it was not surprising that the main inflammation-related transcription factor NF-κB was significantly activated in the TME control cultures compared to the basal control. Confirming numerous previous results (Tyagi et al., 2017; Buhrmann et al., 2020b), treatment with CA lowered the activation of NF-κB (p-NF-κB-p65) similar to the HIF-1α inhibitor. These results were corroborated by a consistent expression of unphosphorylated NF-κB, which served as a control parameter (Figure 6). As increased vascularization enables growth and migration, the metastasis marker β1-integrin was investigated further. Immunoblotting confirmed a migration-promoting effect of the TME, shown by stronger expression in the TME control than in the basal control. At increasing concentrations, CA or the HIF-1α inhibitor inhibited this protein and caused progressively lower expression, suggesting that adhesion and metastasis of the HCT-116 cells were impeded (Figure 6). One of the main goals of tumor therapy is the sustained eradication of cancer cells. Therefore, the expression pattern of apoptosis-related cleaved-caspase-3 was also relevant. Cleaved-caspase-3 was extremely low in the basal control cultures, TME control cultures, and in TME-treated cultures with low CA (1 µM) or HIF-1α inhibitor (1 and 10 µM); however, its expression improved with increasing addition of CA (2 µM) or the HIF-1α inhibitor (20 µM). Finally, clear apoptosis evidence was achieved at CRC cell treatment with 5 µM CA or 30 µM HIF-1α inhibitor (Figure 6).

In total, CA suppressed TME-promoted and HIF-1α-associated neoangiogenesis, vascularization, inflammation, and migration, and elevated apoptosis in HCT-116 cells similar to a HIF-1α inhibitor.

### 3.6 Calebin A suppresses the functional linkage between major tumor-associated transcription factors NF-κB and HIF-1α

**FIGURE 7:**
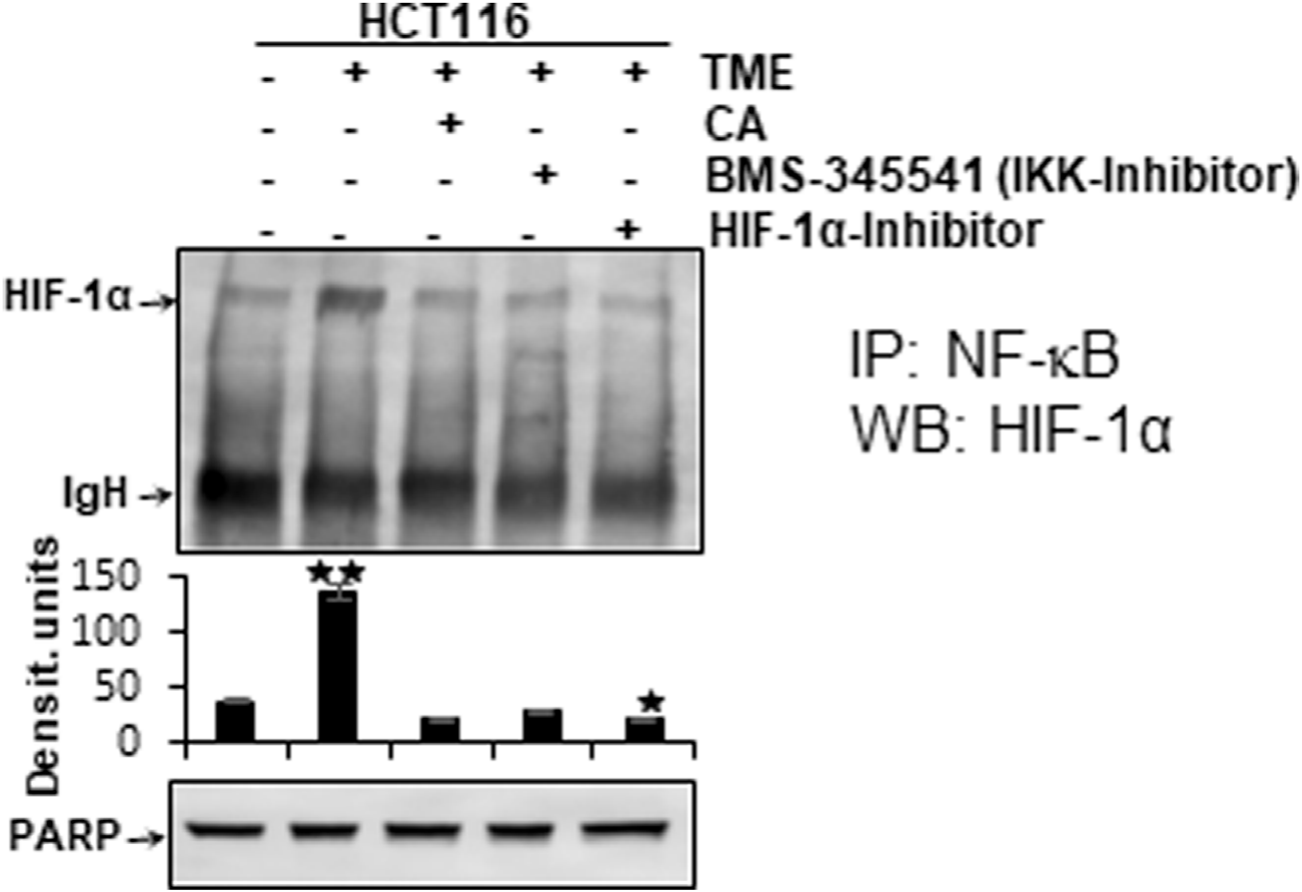
Effect of Calebin A, BMS-345541, or the HIF-1α inhibitor on the functional linkage between NF-κB and HIF-1α. HCT-116 cells were detached from alginate drops, immunoprecipitated (IP) with anti-NF-κB, and immunoblotted (WB) against anti-HIF-1α. Anti-PARP was used as a loading control, and IgH corresponds to the immunoglobulin heavy chain. The X-axis shows CRC cell treatments: basal control, TME control, TME with 5 μM CA, 5 µM BMS-345541, or 20 µM HIF-1α inhibitor. The Y-axis shows densitometric units. *p*-values: *p < 0.05 and **p < 0.01 concerning to basal control.

It has been previously reported that stimulation of both transcription factors HIF-1α and NF-κB induces malignancy, migration, and invasion of tumor cells (Colotta et al., 2009). Moreover, the observation of inflammation-triggered tumor promotion and neoangiogenesis-induced metastatic capacity led to the search for a possible functional link between the major tumor-associated marker proteins NF-κB and HIF-1α in their activated forms. In order to take reciprocity into account, the HCT-116 samples (basal control, TME control, 5 μM CA, 5 µM specific IKK inhibitor (BMS-345541), and 20 µM HIF-1α inhibitor) obtained from 3D alginate cultivation and prepared for immunoblotting were first immunoprecipitated with NF-κB and then immunoblotted with an antibody against HIF-1α (Figure 7). The NF-κB-immunoprecipitated CRC cell samples showed an enhanced HIF-1α expression in the TME control cultures compared with the basal control cultures. This strong angiogenesis induction was significantly limited by treatment with CA, HIF-1α inhibitor, or the IKK inhibitor (Figure 7). The consistent expression of immunoglobulin heavy-chain IgH and the loading control PARP confirmed the balance of the CRC cell samples (Figure 7).

Altogether, the immunoblot demonstrated coupling of HIF-1α and NF-κB, which is interpretable as a functional axis between two tumor-promoting transcription factors. The expression of HIF-1α on this axis was effectively inhibited by CA or the IKK inhibitor. This finding underscores that the effect of 5 µM CA is similarly sufficient to that of the 20 µM HIF-1α inhibitor, suggesting that HIF-1α appears to be a potential subcellular target of anti-tumor effects of CA and resulting in inhibition migration of CRC cells.

## 4 Discussion

CA is a safe natural polyphenol and one of the main components of the rhizome of *Curcuma longa*, which has anti-inflammatory, antioxidant, anti-cancer, and anti-CRC properties (Tyagi et al., 2017; Buhrmann et al., 2019; Buhrmann et al., 2020a; Buhrmann et al., 2021b). Recent works have demonstrated that the use of CA in addition to conventional chemotherapeutic agents efficiently reduces drug resistance (Buhrmann et al., 2019; Buhrmann et al., 2020b; Buhrmann et al., 2021a; Buhrmann et al., 2021b; Brockmueller et al., 2022a; Brockmueller et al., 2023). However, its biological activities and mode of action, particularly as a migration inhibitor for cancer cells, are not yet known.

There is a large body of research data demonstrating that hypoxia is highly expressed in the TME and tumor cells and that it also plays a central role in angiogenesis and malignancy of tumors (Xing et al., 2011; Kumar and Gabrilovich, 2014; Kim et al., 2017; Roy et al., 2020; Zhang et al., 2021). In addition, hypoxia induces the expression of cancer-promoting transcription factors such as HIF-1α, which further induces angiogenesis-promoting growth factors such as VEGF and pro-inflammatory cytokines such as TNF-α and IL-1β, as well as the regulation of immune cells, metabolic signaling, and the development of resistance in various types of tumors in the TME (Kumar and Gabrilovich, 2014; Jiang et al., 2020; Roy et al., 2020). Moreover, the role of the transcription factor NF-κB as a controlling factor in inflammation and metastasis of the TME of CRC is well-studied and established (Oliveira et al., 2015; Tyagi et al., 2017; Buhrmann et al., 2019; Buhrmann et al., 2020a; Buhrmann et al., 2020b), but little is known about HIF-1α in this context. Therefore, in this study, we investigated whether CA exerts its anti-inflammatory and anti-migratory effects via modulation of HIF-1α/NF-κB signaling pathways in CRC-TME, and we compared its anti-carcinogenic effects with a specific HIF-1α inhibitor.

The key insights of these investigations are as follows: CA significantly instigated anti-tumorous properties in CRC cells and was also similarly effective as a HIF-1α inhibitor related to I) reduction of viability, proliferation, invasion, vascularization, inflammation, migration, and II) promotion of apoptosis. Furthermore, III) we show, here, for the first time that the existence of a functional link between HIF-1α and NF-κB in HCT-116 cells has been proven, and IV) that CA shows potential to modulate this newly suspected axis in an anti-carcinogenic manner in CRC.

Our results demonstrated significant inhibition of CRC cell survival, viability, proliferation, and invasion by CA treatment. This basically suggests the anti-tumor modulation of CRC by CA, which is in line with previous findings of a versatile malignancy reduction in the same CRC cell line caused by this phytopharmaceutical (Buhrmann et al., 2021b). The fact that these effects were comparable to those of a synthetic HIF-1α inhibitor led to the assumption of the neoangiogenesis limitation as this is a fundamental requirement for tumor growth and spread. Sufficient vascularization promoted by HIF-1α (Zhang et al., 2015) and the inflamed milieu of a multicellular TME (Buhrmann et al., 2021a) induce EMT, which, in turn, enables tumor metastasis. Most impressively, the migration assay demonstrated successful disruption of this complex process by treatment with CA or a HIF-1α inhibitor.

Furthermore, CA promoted apoptosis similar to the HIF-1α inhibitor shown in the results. In this regard, other research groups demonstrated an apoptosis induction by CA in various cancer cells. The reasons proposed for these polyphenol-associated cell deaths are, for example, a modulation of the mitogen-activated protein kinase (MAPK) pathway in human gastric cancer cells (Li et al., 2008) or an activation of histone acetyltransferase in peripheral nerve sheath tumor cells, which has been confirmed *in vitro* and *in vivo* (Lee et al., 2019). To summarize, we suggest a co-treatment with the natural compound CA could cause tumor cells to die in a similar way to interrupting the blood supply with synthetic medicines.

The cancer-promoting transcription factor HIF-1α is a heterodimeric protein and consists of an oxygen-controlled α-chain and a constant β-chain. Moreover, under hypoxia conditions, the HIF-1α protein is stabilized together with HIF-1β in the nucleus so that the heterodimer can promote the transcription of many HIF-dependent down-stream target genes that mainly promote tumor cell malignancy, including migration (Wu et al., 2015; Zhang et al., 2021). Thereupon, we focused more precisely on HIF-1α, which supports neoangiogenesis and enables the survival of CRC cells despite oxygen-deficient conditions of a carcinogenic milieu (Kim et al., 2017). This tumor-forcing environment, represented by the TME in the previous and this study, is promoted through various pro-inflammatory signaling cascades, particularly those triggered by NF-κB; however, CA significantly blocks and modulates this signaling cascade (Buhrmann et al., 2019). Indeed, NF-κB and the NF-κB signaling pathway, one of the well-researched pro-inflammatory and tumor-promoting transcription factors, have a central function in tumor cell survival, proliferation, angiogenesis, invasion and metastasis (Ahn et al., 2007; Kunnumakkara et al., 2020). For these reasons, we suspected a functional connection between both tumor-associated transcription factors, and we found, for the first time, a functional link between two transcription factors in tandem, and this axis was clearly demonstrated by a co-immunoprecipitation assay. Furthermore, these results are consistent with those of earlier trials demonstrating an NF-κB/HIF-1α/VEGF axis in cancer cells using an *in vivo* angiogenesis assay (Huang et al., 2022). In addition, HIF-1α has been reported to have multiple binding sites for NF-κB p65 (Rius et al., 2008; Raja et al., 2014), highlighting the nature of this link in tumor cells, and our results point out this close relationship between both the transcription factors in promoting CRC cell migration.

We examined further whether CA modulates the HIF-1α/NF-κB tandem and thereby inhibits cancer cell migration. Surprisingly, CA markedly inhibits this functional cooperation between two transcription factors in CRC cells. These results revealed a new and one of the multiple subcellular targets for the anti-tumor potential of CA, thus supporting the multiple modulatory activity of CA against CRC. In addition, we found that TME-promoted expression of many gene end products coordinated by HIF-1α and NF-κB, including VEGF, β1-integrins, and HIF-1α, which are associated with cell migration and metastasis, were downregulated after CA treatment, whereas activation of caspase-3 led to induction of apoptosis (Xing et al., 2011; Kumar and Gabrilovich, 2014; Buhrmann et al., 2019; Kunnumakkara et al., 2020; Roy et al., 2020; Zhang et al., 2021). Indeed, CA has been reported to be able to directly modulate the binding of p65-NF-κB to the DNA promoter (Buhrmann et al., 2020a; Buhrmann et al., 2021a).

Although the suppression of activated NF-κB has been reported in various cancers such as multiple myeloma, head and neck cancer, or CRC (Tyagi et al., 2017), the effect of CA on HIF-1α has not been previously described. The finding that this phytopharmaceutical simultaneously inhibits inflammation, angiogenesis and migration may lead to a new role of CA for future CRC treatment options, particularly in advanced, metastatic, or chemoresistant diseases (Brockmueller et al., 2023).

## 5 Conclusion

The present results demonstrate a CA-associated reduction in cell viability and proliferation, as well as suppression of vascularization, invasion, and migration in HCT-116 CRC cells with simultaneous induction of apoptosis. Interestingly, all findings are associated with the inflammation transcription factor NF-κB and the angiogenesis marker HIF-1α, whose common axis was verified and successfully regulated by CA. This modulatory mechanism of CA is described here for the first time and represents a new research approach for prevention, prognosis, and improved CRC therapy in the future.

## Data Availability

The raw data supporting the conclusion of this article will be made available by the authors, without undue reservation.
